# A pipeline for stochastic and controlled generation of realistic language input for simulating infant language acquisition

**DOI:** 10.3758/s13428-025-02772-6

**Published:** 2025-09-04

**Authors:** Okko Räsänen, Daniil Kocharov

**Affiliations:** Signal Processing Research Centre, Tampere University, Tampere, Finland

**Keywords:** Language development, Computational modeling, Language resources, Speech processing

## Abstract

Computational models of early language development involve implementing theories of learning as functional learning algorithms, exposing these models to realistic language input, and comparing learning outcomes to those in infants. While recent research has made major strides in developing more powerful learning models and evaluation protocols grounded in infant data, models are still predominantly trained with non-naturalistic input data, such as crowd-sourced read speech or text transcripts. This is due to the lack of suitable child-directed speech (CDS) corpora in terms of scale and quality. In parallel, the question of how properties and individual variability in language input affect learning outcomes is an active area of empirical research, underlining the need for realistic yet controllable data for modeling such phenomena. This paper presents a solution to the training data problem through stochastic generation of naturalistic CDS data using statistical models, thereby enabling controlled computational simulations with naturalistic input. We provide a proof-of-concept demonstration of the approach by showing how naturalistic CDS transcripts can be generated with a language model conditioned on recipient information (here, infant age), and how text-to-speech systems can be used to convert the transcripts to high-quality speech with a controllable speaking style. We also conduct modeling experiments with generated speech corpora by varying different aspects of the data, showing how this maps into different learning outcomes, thereby demonstrating the feasibility of the approach for controlled language learning simulations. Finally, we discuss the limitations of using synthetic data in general, and of the present proof-of-concept pipeline in particular.

## Introduction

Research on child language development (CLD) seeks to understand how young children, initially born without language skills, gradually become proficient users of their native language. To do so, infants and toddlers must solve several learning problems, including identifying linguistic patterns in the continuous and variable speech input, mapping these patterns to entities and events beyond language, and learning to understand and produce language as a compositional system capable of expressing an infinite number of messages. Decades of behavioral research on CLD have worked on related questions, such as how infants might segment words from the continuous speech that lacks universal cues for word boundaries (e.g., Lany & Saffran, 2013), how distributional properties of speech might enable development of native language phonemic categorization (e.g., Werker & Tees, 1984), and how words could be mapped to their referential meanings using the statistical regularities connecting heard speech and concurrently observed visual information (e.g., Smith & Yu, 2008). Other studies have examined the role of infant-directed speech (IDS) in CLD (e.g., Soderstrom, 2007) and how infants’ attention and learning relate to the predictability of speech stimuli (e.g., Kidd et al., 2014). Typically, such empirical studies are conducted in highly controlled laboratory conditions with focused research questions, as required by the scientific method.

In order to work toward a bigger picture of CLD, integration of knowledge across individual studies and bits and pieces of empirical data is required. A few high-level theories, such as Word Recognition and Phonetic Structure Acquisition theory (aka. WRAPSA; Jusczyk, 1993), Native Language Magnet Theory Expanded (NLM-e; Kuhl et al., 2008), and a developmental framework for Processing Rich Information from Multi-dimensional Interactive Representations (PRIMIR; Werker & Curtin, 2005), have attempted to summarize the present understanding of early CLD into more comprehensive frameworks, focusing primarily on how infants could bootstrap their phonemic and lexical learning from the raw speech input they have access to. However, none of these frameworks have gained unanimous acceptance in the field. This is largely because they are relatively abstract and only *qualitatively* describe the mechanisms and representations that could be involved in CLD. As a result, *we still have limited understanding of the basic mechanisms and their interactions driving early language acquisition*. This leaves open central questions such as (i) how the discrete, compositional, and hierarchical structure of language (e.g., phonemes, syllables, words, and syntax) can be learned from the variable and continuous acoustic speech input, (ii) how the different aspects of linguistic knowledge interact as a function of a child’s developmental stage, and (iii) how individual differences in language experiences or learning capabilities map into later developmental outcomes and why.

In principle, computational modeling of CLD is a potential solution to the so-called integration problem across empirical findings (e.g., Dupoux, 2018; Cruz Blandón et al., 2023; de Seyssel et al., 2023). This is because computational models can, and must, explicitly address all aspects of the information processing chain from input data to the resulting behavior. By first formulating the theories in terms of high-level computational goals and operations, then implementing them as functional signal processing and machine learning (ML) algorithms, and finally exposing the models to input data comparable to what real infants experience, the ecological plausibility and validity of the underlying theories can be explicitly tested (cf. Marr, 1982).

The existing literature already covers an impressive range of modeling studies addressing topics such as segmentation of speech to phones or syllables (e.g., Scharenborg et al., 2007), word segmentation and discovery from realistic acoustic input (e.g., Park & Glass, 2006; Räsänen, 2011; Vong et al., 2024), learning of phonetic categories (e.g., Schatz et al., 2021), or models of infant articulatory learning (e.g., Guenther, 1995; Rasilo & Räsänen, 2017; Georges et al., 2024; Xu et al., 2024), and many others. Some studies have also addressed the question of potential synergies between different aspects of CLD within one model architecture (e.g., Feldman et al., 2013; Räsänen & Rasilo, 2015; see also Oudeyer et al., 2019, for a review). More recently, so-called zero-resource speech processing research (Glass, 2012; Dunbar et al., 2022) and developments in self-supervised machine learning (SSL) have led to several algorithms capable of unsupervised learning of linguistic structure from speech (e.g., van den Oord et al., 2018; Baevski et al., 2020) or audiovisual data (e.g., Harwath & Glass, 2019). These SSL algorithms have also been adopted as models of infant learners, as their basic operational principles are largely shared with the idea of statistical learning (e.g., Saffran et al., 1996) in infants and predictive processing as a key property of the human brain operation (e.g., Friston et al., 2006; Friston, 2010). For instance, SSL-based models have been successful in learning phonemic and early auditory word forms from speech input (de Seyssel et al., 2023) and in modeling infant-directed speech preference in infants (Cruz Blandón et al., 2023). When paired with audiovisual associative learning from speech and images, SSL models also show emergence of phonemic, syllabic, and lexical representations, including knowledge of word referents (Peng et al., 2023; Khorrami et al., 2023; Harwath & Glass, 2019; Merkx, 2022; see also Chrupała, 2022) and learning from realistic-scale input compared to real infants (Khorrami & Räsänen, 2025).

Overall, the ongoing developments in computational modeling are contributing to our understanding of how language structures could be learned from the noisy and variable sensory speech input that has no discrete or symbolic surface structure—a process dubbed “reverse engineering the infant learner” by Dupoux (2018).

However, the ongoing modeling research activities have a notable shortcoming that also limits the scientific impact of the work: modeling studies are usually conducted on input data that are not representative of infant language experiences in terms of quality or quantity of speech (see, e.g., Cruz Blandón et al., 2023; Dunbar et al., 2022). First, much of infant input comes in the form of child-directed speech (CDS; e.g., Soderstrom, 2007). Second, Coffey et al. (2024) estimated that infants might receive from a few hours up to several thousand hours of speech per year during the first years of life, the estimate varying substantially depending on assumptions about what input counts toward language learning.^1^ This means that up to thousands of hours of speech input would be needed for simulating learning of one child from birth up to 2 years of age.^2^ In comparison, typical datasets used in existing modeling studies consist of read speech in the form of audiobooks (e.g., LibriSpeech with 960 h of audio; Panayotov et al., 2015) or spoken captions of images (e.g., 742 h in SpokenCOCO; Havard et al., 2019; Lin et al., 2014). Naturalistic infant–caregiver interactions at home have also been used (de Seyssel et al., 2023; Lavechin et al., 2023b), but the corpora are also limited in size (e.g., 364 h of speech in the Providence corpus; Demuth et al., 2006). Moreover, such corpora tend to consist of speech from an unrealistically high number of speakers with idiosyncratic speaking styles and recording conditions.

In principle, child-centered long-form recordings from wearable microphones (e.g., Cychosz et al., 2020) could be used instead, but their poor signal quality (Lavechin et al., 2023a; Räsänen et al., 2019) causes problems with automated analysis (e.g., Cristia et al., 2021) and computational modeling experiments (e.g., de Seyssel et al., 2023). This makes it extremely difficult to disentangle the effects of acoustic noise from the real factors of interest in naturalistic versus other speech input. In terms of transcripts, only a tiny proportion of the long-form audios have been manually annotated for what was said, which prevents direct re-synthesis of the speech with good audio quality. Moreover, independently of whether audiobooks or long-form audio is used, the available data are still too limited for modeling the effects of *individual differences* in language experiences at a realistic scale. Finally, the natural long-form audio may contain personal and sensitive data, which is almost impossible to control for. Thus, distribution of the data is limited and strictly controlled by data providers. In contrast, synthetic data does not have this problem, as it can be interpreted as anonymized as long as the generator does not simply memorize the training data.

In an ideal case, the modelers would have access to speech data that (1) are representative in terms of their linguistic and phonetic properties (content, style, proportion of IDS and adult-directed speech [ADS], etc.), (2) are of correct scale (up to thousands of hours; Coffey et al., 2024), (3) are representative in terms of different caregiver voices and their proportions, (4) are appropriate with respect to the simulated infant age, as properties of IDS change with infant development (e.g., Julien & Munson, 2012; Ko, 2012; Kitamura & Burnham, 2003), (5) are of high audio quality (which can be post-processed to reflect various real-world listening conditions as needed), (6) come with annotations (e.g., transcripts and speaker identities), and (7) come with different variants of the dataset that reflect different extralinguistic factors affecting properties of the language heard by young children (e.g., different families and environments) while satisfying all the previous requirements. However, to date, no such data exist, which limits the extent that the effects of infant language exposure can be addressed with computational modeling experiments.

In this paper, we propose a conceptual solution to the lack-of-data problem by presenting a pipeline called *Generator of Infant Language ExperienceS* (GILES) for generation of ecologically representative training data for computational modeling studies. The basic idea is to use large-scale empirical data on real-world infant–caregiver interactions to create stochastic but controllable generators of synthetic speech data, allowing the creation of realistic-scale and age-appropriate speech input for the models while controlling for the factors of interest (e.g., speaking style, vocabulary richness, number of caregivers, amount of background noise). These data will then enable ecologically plausible and replicable experiments with integrated and longitudinal models of CLD, including the possibility of investigating the effects of individual variation in language experience and resulting learning outcomes. By comparing the generated data against the empirical data on infant language experiences, limitations of the synthetic data can be made explicit and corrected.

Besides presenting the basic concept of the GILES, we provide a proof-of-concept study for the pipeline with initial implementations of CDS generation at transcript and acoustic levels. We show how language models (LMs) trained on CDS transcripts can be used to generate new, authentic-looking CDS representative of different recipient ages (Section “CDS transcript generation with an LM”). When paired with a modern text-to-speech (TTS) system (Section “Synthesis of CDS speech with TTS”), these transcripts can then be converted to natural-sounding acoustic speech with a controllable speaking style. We then objectively evaluate the properties of the generated data at linguistic and phonetic levels (Section “Experiment 1: Evaluation of the synthetic data”). Finally, we showcase the use of such data in computational language learning experiments (Section “Experiment 2: Language learning simulations with the synthetic data”), where we use a previously established SSL-based learner model in conjunction with GILES-generated data, and where we control the linguistic content and speaking style of the speech input to observe how these affect the learning outcomes at phonemic and lexical levels.

As our secondary contribution, we provide a systematic analysis of age-dependent changes in linguistic properties of North American English infant-directed speech, as available in the CHILDES (Child Language Data Exchange System) database (MacWhinney, 2000) since this is required as a reference point for analyzing GILES output quality. However, before proceeding to the pipeline description, the next subsection discusses the motivation for using LMs as a means to generate more data for computational experiments.

### On data generation with statistical models

In GILES, the goal is to use LMs for stochastic generation of CDS transcripts and TTS for synthesizing the transcripts to speech. This is in contrast to recent work on text-based models of language acquisition, where researchers have worked toward training LMs on “small” data comparable in size to that available to language-learning children. The typical aim of LM-based studies is to investigate which aspects of grammar LMs can acquire from finite data (Linzen & Baroni, 2021). In this context, a popular resource for text-based CDS data is the CHILDES database of infant–caregiver interactions (MacWhinney, 2000). The North American English section of CHILDES consists of approximately 5 million words, which translates into approximately up to 1 year’s worth of language input to a child (Gilkerson et al., 2017), but of which only a small fraction corresponds to speech directed at infants younger than 12 months. The previous research with CHILDES data has shown that neural LMs can learn grammatical abstractions from the data, either as evaluated from synthetic transcripts sampled from the model (Pannitto & Herbelot, 2020) or by exposing the models to natural language processing (NLP) benchmarks probing various grammatical phenomena (Huebner et al., 2021; Yedetore et al., 2023). Beyond CHILDES, the current best-performing LMs can achieve close-to-human grammatical competence when trained with a comparable number of words to that heard by an approximately 12–14-year-old child (Warstadt et al., 2023).

However, text-based LMs cannot be used as *models* of the first stages of infant language acquisition. This is because infants and young children do not perceive speech in terms of discrete invariant units, such as letters or words (or phonemes), but in terms of complicated and variable acoustic speech where nothing repeats the same way and where the underlying linguistic units and structures are not directly accessible. In fact, how infants manage to acquire useful and sufficiently invariant speech representations, what these representations might be, and at what age they emerge are major modeling research questions in themselves (Dupoux, 2018). Similarly, no computational model to this date has been able to explain acquisition of phonologically invariant speech representations without supervised learning from labeled data.

The existing studies with LMs also do not model the developmental trajectory of child language skills as a function of learner age, even though this would be essential for a comprehensive model of learning (see Cruz Blandón et al., 2023; but see Huebner et al., 2021, for work in this direction). Instead, repeated batch training is typically used to train the LMs with all the available data, after which grammatical benchmarks compare the models’ behavior to adult-like linguistic definitions of appropriate syntax in the language. In reality, the language heard by a learner depends on her linguistic competence, and this competence evolves with age. To model this process in detail, suitable language input data in terms of quality and quantity would be needed throughout the developmental age range of interest. CHILDES is too sparse but also too heterogeneous for the purpose, as it is a collection of various speech datasets collected for different purposes, and with different recording setups in different communicative contexts.

The input a learner receives also varies from one child and family to another, including differences in phonetic properties of the input in addition to the other linguistic levels. How the quality and quantity of language input varies between children and how it affects their learning outcomes is an active topic of research (e.g., Cychosz et al., 2020; Gilkerson et al., 2017). Computational modeling could be a powerful tool for studying individual variability in language learning, but so far there is little work on the topic due to lack of sufficient data, as multiple suitable corpora of a realistic scale would be needed for such simulations. Also, the robustness of developed models should be tested against a variety of alternative yet realistic language exposures to properly validate their feasibility and scalability with realistic input, which is not the case in the present research practice.

All these shortcomings could be addressed if the modeling research community had access to realistic but controlled CDS at a scale comparable to several years of child language input. Then we could start asking questions such as how phonemic perception, word recognition, or syntactic skills emerge from the finite and varying speech input available to children, and regarding the developmental trajectories of the involved capabilities. In addition, such simulations would help to understand how perceptual representations of speech might become invariant enough to connect with the findings from LM-based language acquisition studies, i.e., how infants could learn to represent the incoming speech in terms of discrete categorical entities assumed by modeling studies conducted on symbolic data, such as text or phonemic transcripts (but see also, e.g., Schatz et al., 2021; or McMurray, 2023, for critical views and discussion). By having control over CDS properties as a function of extralinguistic factors, one could also use computational models to study how individual variability in the linguistic and/or acoustic-phonetic properties of input affects the learning process (e.g., linguistic variability and complexity in different families, age-dependent properties of the input, number of speaker voices, speaker intelligibility).

Our present aim is to address the data limitation problem by proposing a pipeline for stochastic generation of representative CDS as a function of extralinguistic factors. We do this by acknowledging that, even though a contemporary text-based LM is not a realistic model of an infant language learner, an LM trained on CDS can be a good model of the *input *at a transcript level without having to worry about the ecological plausibility of the model. At the same time, modern TTS systems can produce high-quality naturalistic speech and are already used for language acquisition simulations (e.g., Havard et al., 2017; Khorrami & Räsänen, 2021). In addition, previous research shows that qualitative findings from phonetic and lexical learning simulations with synthetic speech generalize to real speech (Khorrami & Räsänen, 2021). Hence, we show that an LM can be trained to generate authentic-looking but novel CDS utterances so that the generation is modulated by extralinguistic factors, here using the recipient child’s age as a proof of concept for the controllability, which can then be synthesized into speech of varied speaking styles with TTS.

We acknowledge that the creation of training data with a statistical model to be used as input to train (statistical) learner models comes with potential caveats, and it may also seem circular or counterintuitive at first. The key insight is that the training of the data generators (LM and TTS) can utilize additional data, information, and learning mechanisms that are not available to infant learners that we are ultimately interested in modeling. These include supervised training with ground-truth information and symbolic representations that simplify the learning process substantially, as both the LM and TTS make use of a transcript-level linguistic form corresponding to the observable speech acoustics. They can also use arbitrarily large training data to learn general or CDS-specific aspects of language and speech. In addition, they can use iterated batch training, and can apply other steps for model selection, data structuring, and output post-processing, and may use any available metadata that can support the training process. None of these aspects is part of realistic simulations of infant learners dealing with finite and incrementally appearing input—input that comes in the form of acoustic speech that lacks universal cues to the underlying linguistic structure. When properly combined, finite (but potentially large) training resources could be used to create infinite but controlled variation in the generated outputs in the same way that large LMs are nowadays used to create infinite forms of new linguistic content from finite (but large) training data. Overall, this creates a highly asymmetric situation in terms of learning in the generator versus learning in the infant model, where many of the phenomena we want to understand for infant learners (phonemic and lexical bootstrapping, beginnings of syntax, etc.) are nearly trivial to solve for modern ML with access to supervised training, symbolic forms of language, and large training data, but are not well understood in the case of learning from the acoustic speech input at a realistic scale (cf. Dupoux, 2018).

We also address the issue of variability by carefully measuring and ensuring that the generators create linguistic variability beyond their training data. Through systematic and rich measurement of the created data properties, the approach additionally enables full transparency regarding the limitations of the given simulations—an issue which is actually not well understood even for standard learning simulations with real but non-naturalistic speech inputs, such as crowd-sourced audiobooks or small-scale speech corpora. Also, note that the use of artificial language data has been, and still is, a method for studying language learnability in linguistics, such as in the context of grammatical inference from symbolic input (Gold, 1967; Dupre, 2024). Our current work adopts the same basic idea of having (statistically) controlled data, but goes beyond the classical practice by proposing the use of artificial *but representative and varied* speech data *at a realistic scale* to study potential mechanisms that could enable early stages of infant language learning from finite input data.

## Generator of infant language experiences (GILES)

The general aim of GILES is to enable controlled creation of naturalistic CDS (and ADS to an appropriate extent for overheard speech) at a realistic scale in order to facilitate computational studies of language learning from acoustic speech input. The idea is to use the existing empirical data on infant language experiences, such as transcripts of CDS to capture the linguistic properties of the input and acoustic CDS for prosodic modeling, in order to create a system that can then be sampled for speech data at a desired scale.

Moreover, the generating processes are conditioned by *extralinguistic factors* that can affect the content and style of the speech, which are also grounded in empirical datasets or results from infant studies. As an example, infant age is a central factor that should be incorporated in developmental simulations, as CDS undergoes various linguistic and phonetic changes as the child develops (e.g., Soderstrom, 2007; Julien & Munson, 2012; Ko, 2012; see also Section"Text analysis"). More generally, there is empirical variation across infants’ language environments (~families) in terms of what kind of language is used (e.g., vocabulary size and syntactic complexity) and how often, the number of voices heard by the infant, the proportion of CDS to ADS, and many other factors. The idea in GILES is to incorporate different factors into the generation process, thereby enabling controlled generation of different language experiences. To operate the system, the user is expected to define (or sample from an empirical distribution) values for the factors of interest, including how many hours of speech are to be generated and how the factors might change during the speech timeline.

**Fig. 1:**
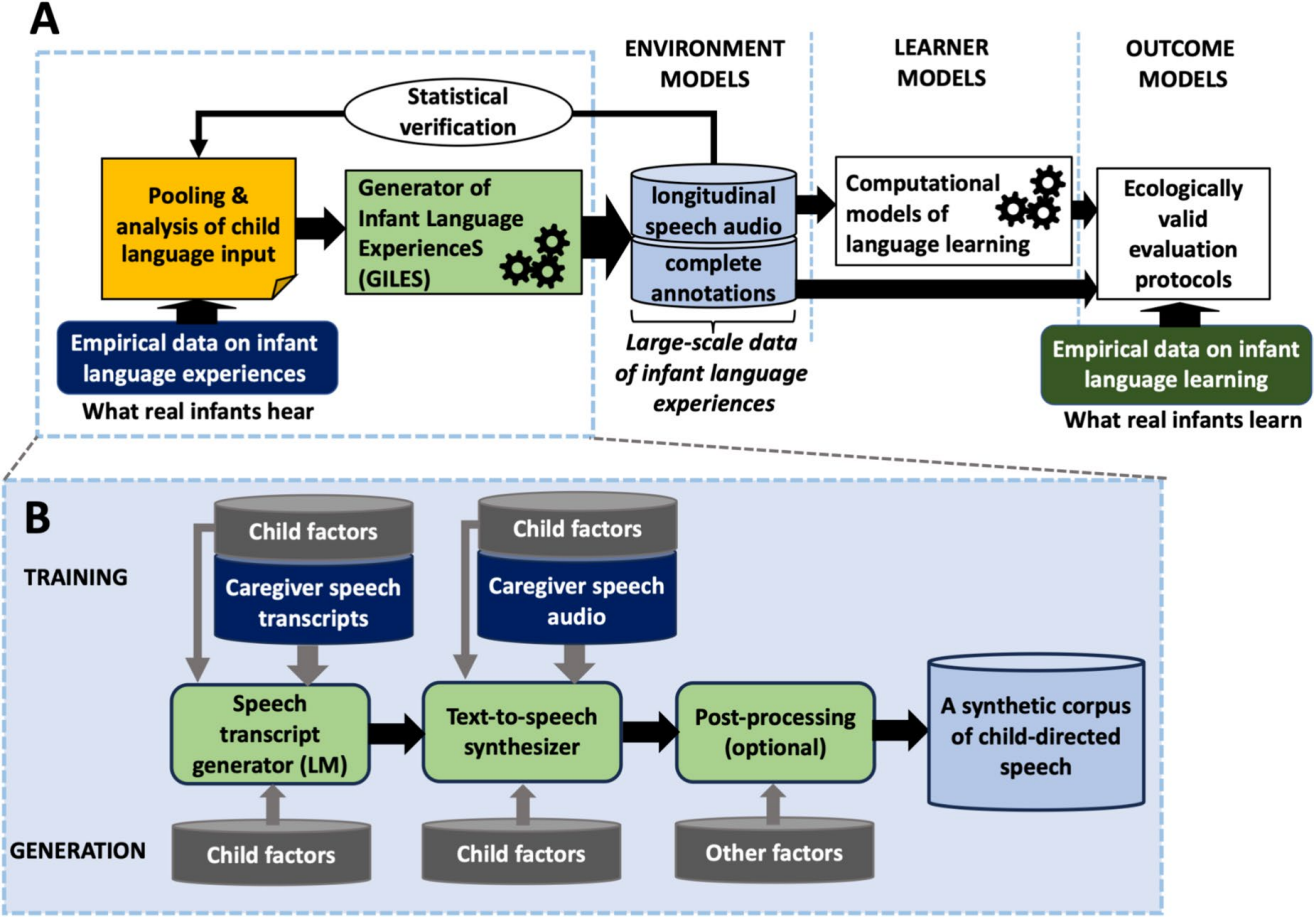
**A** Schematic view of a standard computational modeling pipeline for studying infant language acquisition, but where GILES replaces prerecorded speech corpora as the environment model (see de Seyssel et al., 2023, for discussion on the environment, learner, and outcome models in simulations). **B** A high-level overview of the GILES structure. Empirical data on infant language exposure and associated metadata (e.g., child age, family socioeconomic status, normative/non-normative), aka “factors,” are used to train a generator of caregiver speech transcripts and a speech synthesizer capable of representative speaking styles. At generation time, desired child factors and simulation-specific factors (simulated age range, potential noise levels, etc.) are used to drive the generators to obtain a corpus of speech representing language experience of a virtual learner

Figure 1A shows the general framework for computational modeling of language learning and how GILES fits into the framework. Figure 1B shows the conceptual overview of GILES itself.

From a technical perspective, GILES consists of two key components (Fig. 1B): an LM for generating text-level transcripts of caregiver speech toward infants, responsible for capturing the lexical and syntactic properties of CDS, and a TTS model for synthesizing the generated transcripts as acoustic speech. These modules can be trained independently using different data sources, as long as the training data contain representative language input to those infant populations whose learning one then wishes to model with GILES-generated data (e.g., a particular caregiver dialect or audience group). If enough data exist for the target domain, both modules could be trained directly on those data only. However, in practice, the existing resources for CDS transcripts or high-quality speech audio recordings are still sparse. More generic training with a broader set of language resources, combined with appropriate generator conditioning over factors of interest (e.g., explicitly conditioning the generator with dialect and audience information), is likely to produce higher-quality results while allowing customization of the “target audience” for the input. This is because modern generative machine learning models have the capacity to harness what is common to different subsets of the training data (e.g., to learn the general structure of language from large data) and then use that as the basis for learning specific characteristics of subsets from smaller data (e.g., learning how dialect impacts language properties).

In addition to the LM and TTS, the pipeline consists of optional post-processing techniques to improve the quality of the generated data and/or to manipulate properties of the data to study the effects of factors that are difficult or unreasonable to learn as a part of the LM or TTS training. As an example of the former, NLP techniques with empirical word frequency statistics could be applied to counter the potential reduction of lexical variability caused by statistical modeling (cf. Dohmatob et al., 2024; see also Discussion). As for the latter, the produced data could be, for instance, augmented with various levels and types of overlapping or interwoven environmental noises to study their impact on the learning process (cf. de Seyssel et al., 2023). In addition, voice conversion algorithms or vocoder-based speech modifications could be used to extend the original voice repertoire of the TTS system without requiring these voices in the CDS TTS training data.

**Fig. 2:**
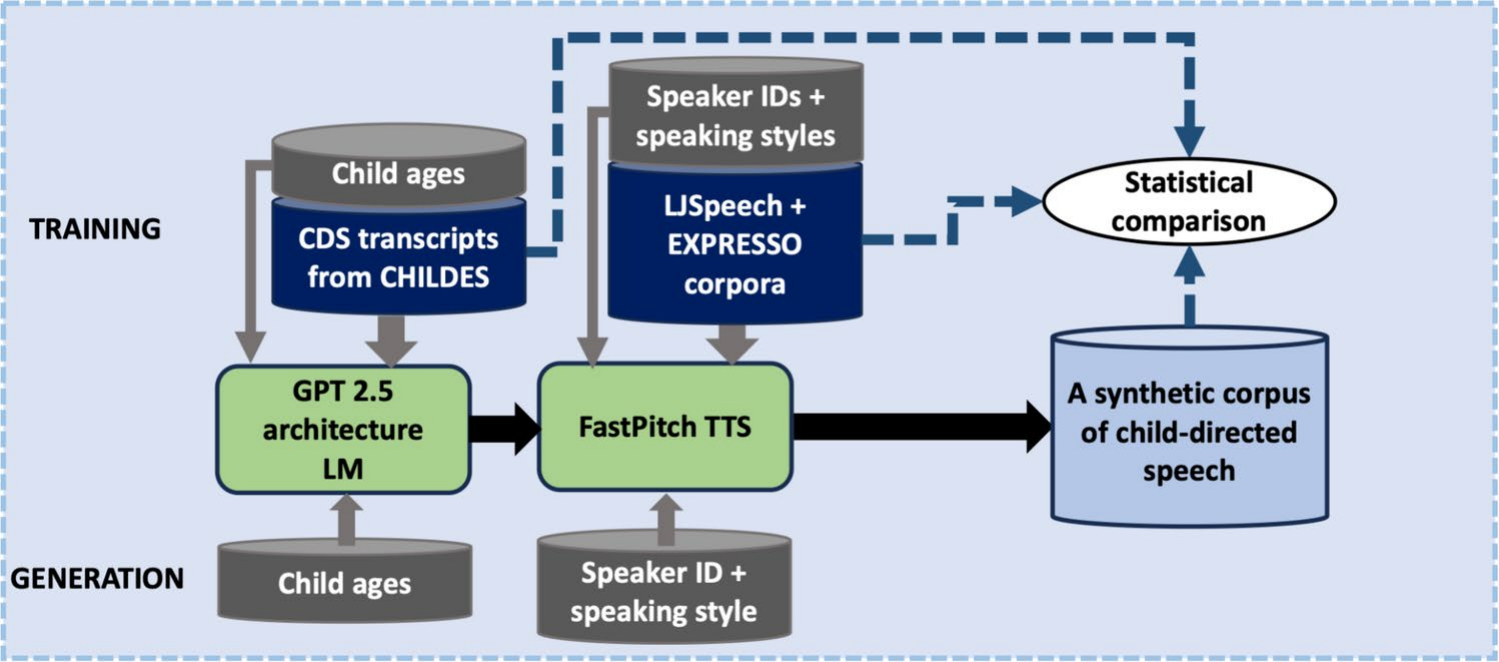
Proof-of-concept implementation of GILES used in the present study

Obviously, developing a representative and comprehensive model of speech input heard by infants in different circumstances, and at both linguistic and acoustic levels, is a massive and incremental research effort in itself, and therefore beyond the scope of this paper. In the present proof-of-concept study of GILES (Fig. 2), we will limit child factors to the age of the recipient child during the speech transcript generation. This exemplifies the approach where we can use a larger set of CDS text data to learn general properties of CDS (and English in general) while also enforcing the model to learn how the “general” CDS then varies with recipient age. In addition, we will use two distinct speaking styles for the TTS, neither of which accurately represents CDS prosody, but which demonstrate the controllability of the acoustic part of the pipeline. This is due to the lack of an existing TTS system capable of CDS style and lack of high-quality naturalistic CDS data at a required scale to train a TTS system from scratch. Due to lack of suitable training data, age-dependent CDS TTS is also not currently technically feasible.

### CDS transcript generation with an LM

An LM is employed to generate transcripts that simulate age-appropriate CDS of caregivers toward their children. The current version of GILES uses a transformer-based deep neural network that follows the basic GPT-2 architecture (Radford et al., 2018) with a decoder module only. Input WordPiece tokens are first passed to 512-dimensional embedding layers for word tokens and token positions, where token and positional embeddings are combined through summation. For age conditioning, a separate embedding layer converts the recipient infant age (scalar) into a 512-dimensional embedding using a feed-forward layer with rectified linear unit (ReLU) activations. This age embedding is then concatenated in front of the positionally encoded word token embeddings. The sequence of embeddings is then processed by five transformer blocks, each with 512-dimensional latent and output layers, eight self-attention heads, and a dropout rate of 0.05 between the blocks. The final layer is a softmax layer that maps the output from the last transformer block into a posterior probability distribution across the token vocabulary.

To demonstrate the system in proof-of-concept experiments in this paper, we trained the LM using the North American English corpora of AO (age-ordering)-CHILDES (Huebner & Willits, 2021), as accessed using the childes-db interface (Sanchez et al., 2019; database version 2021.1). From now on, we will use the term *utterance* for the basic unit of transcribed speech in both the training and generated data, as delimited by full stops in the CHILDES transcripts. Spontaneous speech transcripts from CHILDES have inconsistent punctuation marking due to the high complexity involved in punctuating spontaneous speech. Thus, we preprocessed the training data by removing all punctuation marks, lowering the case, and using only a full stop to mark the end of an utterance. All transcripts were converted to lowercase using whitespaces as word delimiters. Transcripts of utterances by mothers and fathers were assigned to 3-month age bins, centered at 3, 6, 9, …, 84 months, for age-dependent training, evaluation, and model age-conditioning purposes. After exclusion of utterances without child age information or transcription markers for incomprehensible speech, this resulted in transcripts with a total of 3.82 million word tokens and 862,992 utterances. The BERT WordPiece tokenizer (Devlin et al., 2019) with a vocabulary size of 8,000 tokens was then used to tokenize the resulting word strings.

For the LM training, data from all the age bins were used except for the 57-month age bin, which was used for model validation. All the training tokens were concatenated into a long string, which was then split into a total of 51,740 samples of 100 tokens (approximately 74 words) each. The LM input sequence length was set to 100 tokens, and the model task was to predict the next token for each of the input tokens, as implemented by causal masking of the self-attention layers of the network. The model was trained with categorical cross-entropy loss using minibatches of 64 samples, the Adam optimizer, and a learning rate of 0.0001. Validation loss was used for early stopping of training with patience of 15, and the best model was selected based on the validation loss.

After the training process, transcripts generated from the resulting LM should simulate generic CDS with its properties learned and generalized across the heterogeneous collection of CHILDES corpora. In a similar manner, the age-dependent changes in the generated CDS due to age conditioning should reflect general tendencies of CHILDES data available for different child ages, as data from several different corpora were always available for each age bin (mean 10.6, min 2, and max 17 corpora per bin). In Section"Results of the transcript analysis", we analyze how well these characteristics were learned by the model. In Appendix 1, we present some LM outputs conditioned with different target child ages.

### Synthesis of CDS speech with TTS

The purpose of the TTS block is to convert the CDS transcripts into acoustic speech, ideally representing natural speech as accurately as possible. GILES uses FastPitch TTS extended with style conditioning (Łańcucki, 2021), which is a fully parallel text-to-speech model based on FastSpeech (Ren et al., 2019) conditioned on fundamental frequency contours. A pretrained HiFi-GAN vocoder was used to convert mel-spectrograms produced by FastPitch into speech signals (Kong et al., 2020). The style conditioning was implemented the same way as speaker conditioning in the original FastPitch architecture.

Two datasets were used for the TTS training: the LJ Speech Dataset (Ito & Johnson, 2017; hereafter, LJSpeech) and the EXPRESSO Dataset (Nguyen et al., 2023). The LJSpeech consists of short audio clips of a single female speaker of North American English reading passages from nonfiction books. Clips vary in length from 1 to 10 s and have a total duration of approximately 24 h. EXPRESSO contains both expressive read speech (eight styles) and improvised dialogues (26 styles). The dataset includes speech by four North American English speakers (two male, two female), with a total of 45.9 h (11.5 h read, 34.4 h improvised). The read speech section of EXPRESSO includes provided transcriptions.

The LJSpeech did not require any preparation and was used as it was. EXPRESSO recordings were segmented into shorter audio clips using a Python script provided as a part of the EXPRESSO dataset, and then the dialogue recordings were transcribed into text using the large-v3 model of the Whisper toolkit by OpenAI (Radford et al., 2023).

We used data from four speaking styles of EXPRESSO in our experiments. *Default* and *narration* speech styles were pooled together and labeled as *neutral* style. *Child-directed* and *animal-directed* speech styles were pooled together and labeled as *CDS* style. This was done because EXPRESSO contains only 38 min of child-directed speech and 32 min of animal-directed speech. Whereas child- and pet-directed speech are prosodically similar to each other and distinct from ADS (Burnham et al., 2002), they do differ in terms of linguistic content. For instance, pet-directed speech contains more imperatives and fewer questions and declaratives than CDS (Mitchell, 2001), but this should not be an issue for our approach, where the linguistic content is created by an LM independently of the TTS prosody. Both child-directed and animal-directed speech was acted. All recordings from the LJSpeech were assigned to the neutral style. Thus, we had five speakers and two styles in total. Each audio clip was labeled with a speaker and a style. Given the non-naturalistic nature and limited size of the CDS training data in EXPRESSO, we acknowledge that the present CDS style does not fully represent naturalistic CDS found in real-world settings. Instead, we refer to this style as prosodically “CDS” to contrast it with the neutral style, while leaving the development of a full-fledged, high-quality naturalistic CDS speech synthesizer for future work.^3^

The TTS model was trained for 1,500 epochs with two NVIDIA V100 graphics processing units (GPUs) using default hyperparameters of FastPitch, while using speaking styles and speaker identities as conditioning information.

## Experiment 1: Evaluation of the synthetic data

The aim of the first experiment was to evaluate how well properties of the CDS produced by GILES match that of real speech. To do this, we first generated a dataset of age-dependent transcripts and corresponding acoustic speech, and then evaluated the output in terms of linguistic structure and prosodic properties, as detailed in the following subsections.

### Data generation

The LM was used to create CDS transcripts for ages of 6, 9, 12, 15, 18, 21, 24, 36, and 48 months using the corresponding age as the conditioning variable for the model. For each bin, we created 2,000 samples of contiguous text strings, with a seed prompt of 1–4 contiguous tokens randomly sampled from the original training data for each string. Starting from the seed, the token-by-token generation was run by sampling from the posterior distribution of the next token using a temperature of 1 (the original posterior), limiting the choices to the top 500 most likely tokens, and adding the sampled token to the generated string. Generation was continued until a total of 60 tokens was reached, after which the last utterance was discarded unless it finished with a full stop. The first utterance containing the initial seed was also discarded. After converting the BERT-encoded tokens back to words, the process resulted in synthetic transcripts with a total of approximately 75,000 words generated per age bin.

The generated transcripts were then synthesized into speech using the LJSpeech female voice, as this voice had the best intelligibility compared to the EXPRESSO voices (evaluated in terms of Whisper automatic speech recognition [ASR] system word error rate). To enable comparisons, we synthesized two different versions of the transcripts by conditioning the TTS model with the two speaking styles: neutral and CDS.

To further facilitate evaluation of GILES, we also generated synthetic speech datasets using

1. Book contents from the train-clean-100 split of the LibriSpeech corpus (Panayotov et al., 2015) and
2. Original CHILDES transcripts of real CDS addressed to infants.

### Text analysis

To assess the lexical and syntactic properties of the generated data, several metrics were calculated for each age bin of the generated data and compared to the original CHILDES transcripts for the corresponding infant ages. The metrics included the following:

- Mean utterance perplexity
- Lexical richness:
  - type-to-token ratio (TTR) of lexemes
  - rate of different part-of-speech (POS) categories
  - POS-dependent TTR of lexemes
  - lexical divergence—frequency distributions of lexemes
- Syntactic complexity:
  - mean utterance length (in words)
  - rate of utterances containing dependent clauses
  - mean height of syntactic tree
  - mean number of dependents per word
  - average dependency distance

Perplexity was calculated as a holistic measure for lexical and syntactic acceptability and diversity using a pretrained LM model of English language, i.e., GPT-2^4^ by OpenAI. For further syntactic and lexical analysis, all the texts were POS-tagged and parsed. The Stanza toolkit (Qi et al., 2020) was used to perform data tokenization, POS tagging, lemmatization, and syntactic parsing while treating utterances as sentences. This process resulted in grammatical tagging and tree representations following the Universal Dependencies formalism. The utterance transcriptions with no sentence splitting were regarded as single sentences.

TTR was calculated as a general measure of lexical richness. The rate and TTR of different POS words were used for analyzing lexical richness in more detail. We analyzed both open and closed word classes. Open word classes included adjectives (ADJ), adverbs (ADV), nouns (NOUN), verbs (VERB), and interjections (INTJ). Closed word classes included pronouns (PRON), determiners (DET), and conjunctions (CONJ). The conjunctions class was a joint class of coordinating conjunctions (CCONJ) and subordinating conjunctions (SCONJ).

Lexical divergence was measured to determine whether relative lexeme frequencies, and consequently their frequency ranks, were comparable between the synthetic and original data. Lexical divergence was calculated against the CHILDES 60-month-old bin. This was done by taking a fixed-size sample from both the generated transcripts and the CHILDES 60-month-old bin, calculating frequencies of all lexemes that occur at least twice in the data, and then calculating Jensen–Shannon divergence between the frequency distributions of the two samples (where a lower value means more similar distributions).

Mean utterance length was used to express the general syntactic complexity of an utterance. The average dependency distance (Oya, 2011), the rate of utterances consisting of at least two clauses, the mean height of the syntactic tree, and the mean number of dependents per word were used to analyze syntactic complexity in more detail. These metrics were obtained by the analysis of the syntactic trees of the utterances.

Since some of the metrics can be affected by the dataset size (e.g., TTR; see, e.g., Montag et al., 2018), we calculated all the metrics for 100 equal-size random subsamples of the data when sampling with replacement. For each sampling, 10,000 words were randomly sampled for the measures of lexical richness, and 1,000 parsed utterances were sampled for measures of syntactic complexity. For perplexity evaluation, we sampled 100 strings of at least 50 words so that the strings consisted of contiguous complete utterances.

To ensure that the model was not simply memorizing the training data, we also calculated the proportion of generated utterances (word strings without the final stops) that never occurred as (sub)strings in the training data. The proportion is reported relative to utterance length (in words), as shorter utterances are likely to recur more often.

### Prosodic analysis

The most widely acknowledged universal phonetic properties of CDS as opposed to ADS are the higher and more variable pitch, slower tempo, and longer pauses and vowels (Soderstrom, 2007). The acoustic properties of speech sounds in CDS are disputed—for example, some researchers have shown stretching of the vowel space in CDS (Kuhl et al., 1997) while others showed its shrinking (Benders, 2013), and some have shown that the expansion of vowel space in CDS did not lead to better vowel differentiation (Miyazawa et al., 2017).

In the current experiment, we measured the main universal properties of speech, highlighted previously for the opposition of CDS and ADS, i.e., fundamental frequency (*F*_0_) height and variability, speech tempo, and vowel duration. We did not evaluate pausing, as long pauses are not present in the TTS training datasets and therefore do not appear in the output.

The text–speech alignment was produced by the Montreal Forced Aligner (McAuliffe et al., 2017) using a US English pronunciation dictionary and acoustic model v.3.1. Random samples were selected from each synthetic dataset to calculate acoustic features. *F*_0_ was calculated by the probabilistic YIN (pYIN) algorithm (Matthias & Dixon, 2014) using the librosa toolkit.^5^ The *F*_0_ values for voiceless consonants were linearly interpolated, and the melodic contour was smoothed using Golay–Savitzky filtering with third-order polynomials in five sample windows (Savitzky & Golay, 1964). Pitch height was acoustically estimated by the *F*_0_ value of the accent of the first word in the utterance, as it is less dependent on the content of the utterance than the *F*_0_ mean within an utterance (Yuan & Liberman, 2014; Kocharov et al., 2015). Pitch variability was acoustically estimated by the standard deviation of *F*_0_ within an utterance in semitones relative to the mean *F*_0_ within the utterance. Speech tempo was estimated by the number of vowel sounds per second as a proxy of the number of syllables per second. Vowel duration was calculated based on the aligned transcripts.

To compare synthetic speech to real speech, an additional set of original speech samples from LJSpeech was used. The total duration of speech samples from each dataset was 20 h.

### Results of the transcript analysis

**Fig. 3:**
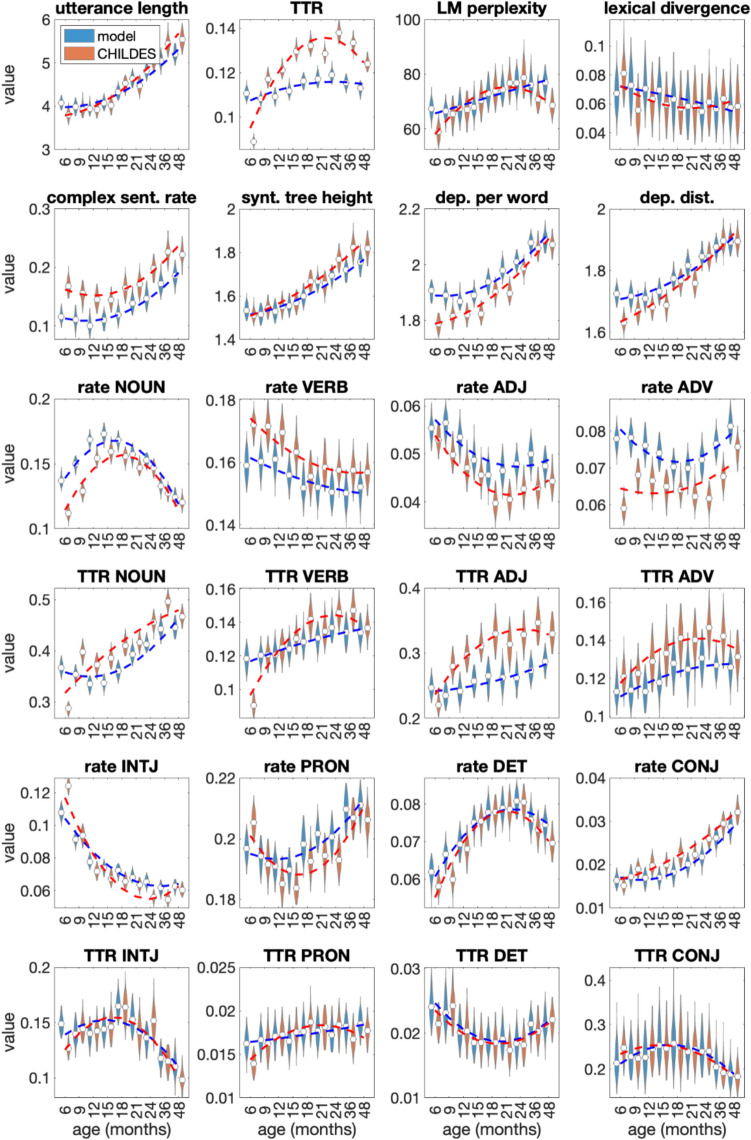
Statistical characteristics of the generated (blue) and original (red) CDS transcripts as a function of simulated/real infant age (*x*-axis), as measured across various aspects of lexical and syntactic properties of utterances in the transcripts. Violin plots show the distributions of measures from 100 random sub-samplings, and dashed lines denote second-order polynomial fits to the corresponding measurements. TTR refers to type-to-token ratio of words and rate to the proportion of given part-of-speech words compared to all words. NOUN = nouns, VERB = verbs, ADJ = adjectives, ADV = adverbials, INTJ = interjections, PRON = pronouns, DET = determines, and CONJ = conjunctions. Note the nonlinear spacing of age on the *x*-axes

Figure 3 shows the evaluation outcomes for the original CHILDES- and GILES-generated transcripts as a function of real/simulated child age. The linguistic analysis of CDS in CHILDES reveals the age-dependent increase in language complexity that qualitatively aligns with earlier research on change in CDS with age (Soderstrom, 2007): caregiver vocabulary becomes richer (increasing TTR of content words), syntax becomes generally more complex (higher perplexity, more dependencies per root), and the utterances become longer. The increase in perplexity reflects the overall increase in linguistic complexity.

The analysis of POS-specific occurrence rates and POS-specific TTRs reveals their negative correlation: the smaller the rate, the larger the TTR and vice versa, i.e., the richness of the given POS vocabulary depends on the given POS rate. Exceptions to this are nouns and interjections. Nouns show a constant increase in TTR independent of their relative frequency in speech; thus the variability of noun lexemes increases relative to their use. The proportion of interjections decreases systematically. The TTR of interjections starts to decrease after an age of 15 months, suggesting that caregivers still use the same vocabulary of interjections while reducing their overall number in speech.

The syntactic metrics show an almost linear increase with the age of a target child, indicating a gradual increase in syntactic complexity. Within the target child age range from 0;6 to 4;0, a significant increase is found in syntactic tree height (20%), the distance between syntactically connected words (16%), and the average number of dependent words (17%). As a result, the utterances increase in length by up to 50%, from four to almost six words on average.

A key observation is a nonlinear U-shaped curve for the majority of both syntactic and lexical metrics, typically with a turning point around 15 to 21 months of age. This nonlinearity is stronger for some metrics, such as the rate of nouns, and very subtle for others such as the rate of complex sentences or the rate of verbs. While this could partially be an artifact of the heterogeneous corpora included in CHILDES, we hypothesize that it may also reflect the rapidly evolving lexical and other language skills of infants around this age range (cf., e.g., the MacArthur-Bates Communicative Development Inventories [CDI] vocabulary norms in WordBank; Frank et al., 2016). Assuming that caregivers adapt their speech to their infants’ developmental level, the temporary drop in the proportion of adjectives and pronouns might be a trade-off for rapidly increasing naming of objects and events to the learner. In fact, if single-word utterances are excluded from the analysis, the complexity measures (perplexity, number of dependencies per root, utterance length) still reflect slight U-shaped behavior, indicating that the more complex multi-word utterances used during the pre-linguistic stage become simpler around 12 months of age before gradually growing toward more complex adult-like language (not shown separately).

As for the synthetic data, the LM appears to qualitatively replicate the basic age-dependent patterns of the original data, albeit with some minor differences. The synthetic texts have smoother changes in statistics as a function of age than those in the original CHILDES (see, e.g., rate and TTR of interjections and pronouns), where some of the sudden changes in CHILDES may originate from the heterogeneity of the included corpora for different age bins (e.g., TTR of nouns). There are some outlier values for certain ages in the CHILDES data, such as TTR of interjections at 6 months. In this case, the generated data have fewer outliers.

To analyze the results quantitatively, we calculated a two-tailed Student *t*-test between the measure distributions of GILES-generated transcripts against original CHILDES for each age bin. The test showed a significant difference (*p* < 0.05) between nearly all pairs of compared conditions except for rare cases (e.g., the LM perplexity at the age of 12 and 18 months). However, with such a large sample size, the statistical difference is not as interesting as the effect size, which can be seen as the divergence of the distributions. For this reason, Table 1 reports the mean effect sizes across all age bins using Cohen’s *d* (Cohen, 1988). The table also reports relative absolute differences (%) of the means of the measures, as the relative error provides the most intuitive measure of how much the generated data deviate from the real CHILDES transcripts. Table 2 reports the metrics for each age bin, averaged across all the linguistic measures.

**Table 1:** Mean effect sizes between linguistic measures calculated across all age bins: Cohen’s *d* and relative absolute difference between distribution means (%)

Linguistic measure	Cohen’s *d*	Relative absolute difference (%)
Utterance length	1.74	3.99
TTR	7.89	12.56
Utterance perplexity	1.15	5.02
Lexical divergence	0.72	12.45
Rate of utterances containing dependent clauses	4.00	24.83
Height of syntactic tree of the utterance	1.62	2.67
Mean number of dependents per word	2.63	2.93
Average dependency distance	1.67	1.99
Adjective rate	2.61	11.49
Adverb rate	4.07	15.12
Conjunction rate	2.15	12.72
Determiner rate	0.95	3.40
Interjection rate	2.77	9.26
Noun rate	3.71	9.17
Pronoun rate	1.54	2.84
Verb rate	2.23	4.75
Adjective TTR	3.88	17.21
Adverb TTR	1.79	9.33
Conjunction TTR	0.33	4.40
Determiner TTR	0.46	4.12
Interjection TTR	1.08	6.56
Noun TTR	4.58	11.53
Pronoun TTR	0.80	5.62
Verb TTR	1.75	7.46

**Table 2:** Mean effect size between age bins calculated across all linguistic measures: Cohen’s *d* and relative difference between distribution means (%)

Age bin (months)	Cohen’s *d*	Relative difference (%)	Number of tokens in the training dataset
6	4.16	14.23	24,040
9	2.17	8.18	175,138
12	2.08	7.90	270,097
15	2.24	7.80	331,665
18	2.10	7.87	287,744
21	1.90	6.71	379,910
24	2.29	8.22	337,535
36	2.43	8.66	285,021
48	1.69	5.95	90,579

The mean Cohen’s *d* value over all ages varies for different linguistic measures from 0.33 (TTR of conjunctions) to 7.89 (TTR). The mean relative difference varies from 1.99% (average dependency distance) to 24.83% (rate of utterances containing dependent clauses). Notably, the 6-month age bin diverges from the rest of the age bins, having the largest difference between original CHILDES and GILES-generated transcripts, being almost twice as large as the other age bins (see Table 2). This might be due to the small number of training data (original CHILDES data) for this age bin (~24,000 tokens) compared to, e.g., the second-smallest age bin of 48 months (~90,000 tokens). The lack of data might have an effect on both the training of the LM and the representativeness of the reference CHILDES data.

Overall, despite the statistically significant differences, the relative errors in the generated versus real data are relatively small for the majority of the linguistic measures. The syntactic properties of the synthetic transcripts are very similar to those of the original data (Fig. 3), including the syntactic tree height, dependency distance, and number of dependent words per each word in a sentence. The lexical properties of the synthetic transcripts exhibit some differences from those of the original data, as observed in the rate and type-to-token ratio (TTR) of content words (nouns, verbs, adjectives, and adverbs), while the distribution of frequent non-content words (interjections, conjunctions, pronouns, and determiners) is well modeled. This reflects the smaller vocabulary size of the generated texts, as rare words from the original data are not produced by the model when generating synthetic transcripts (see also Dohmatob et al., 2024).

**Fig. 4:**
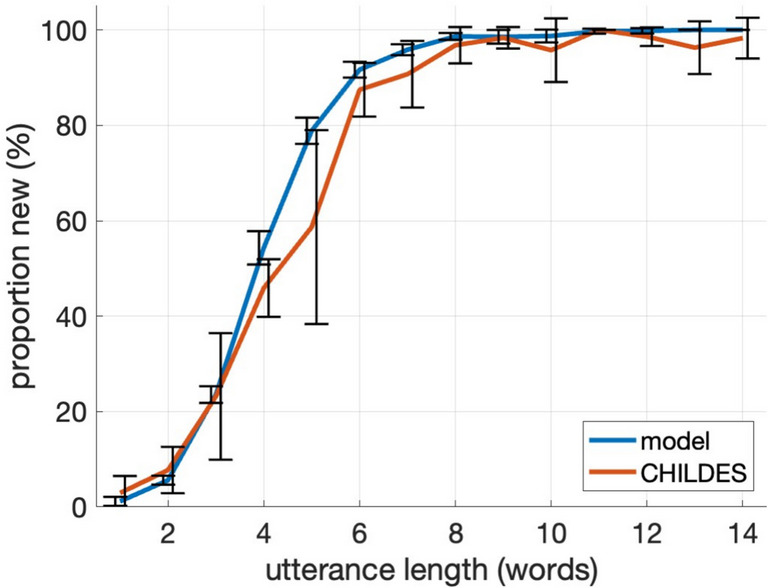
Proportion of previously unattested utterances. Model: utterances that never occurred in the CHILDES training data. CHILDES: utterances that only appear once in CHILDES. Vertical black bars denote standard deviation at each utterance length

Figure 4 shows the proportion of synthetic utterances that never occur in the training data as well as the proportion of non-repeating utterances in CHILDES. Approximately 60% of the synthetic four-word utterances and nearly all long utterances are completely new, thereby confirming that the model has not memorized the training data. Instead, it is using compositional knowledge to create new messages while following the statistics and linguistic characteristics of the real CDS (cf. perplexity and grammatical measures in Fig. 3). Moreover, the rate at which the model generates completely novel utterances is similar to the proportion of unique utterances in CHILDES. This shows that the model is comparable to real speakers in generating new, unattested messages from a finite vocabulary to which they have been previously exposed, introducing linguistic variability to the data that did not exist in the original CHILDES. We also manually analyzed the unique words or utterances generated by the model and those in CHILDES, and we did not find obvious qualitative differences between the two sources.

### Speech analysis results

**Fig. 5:**
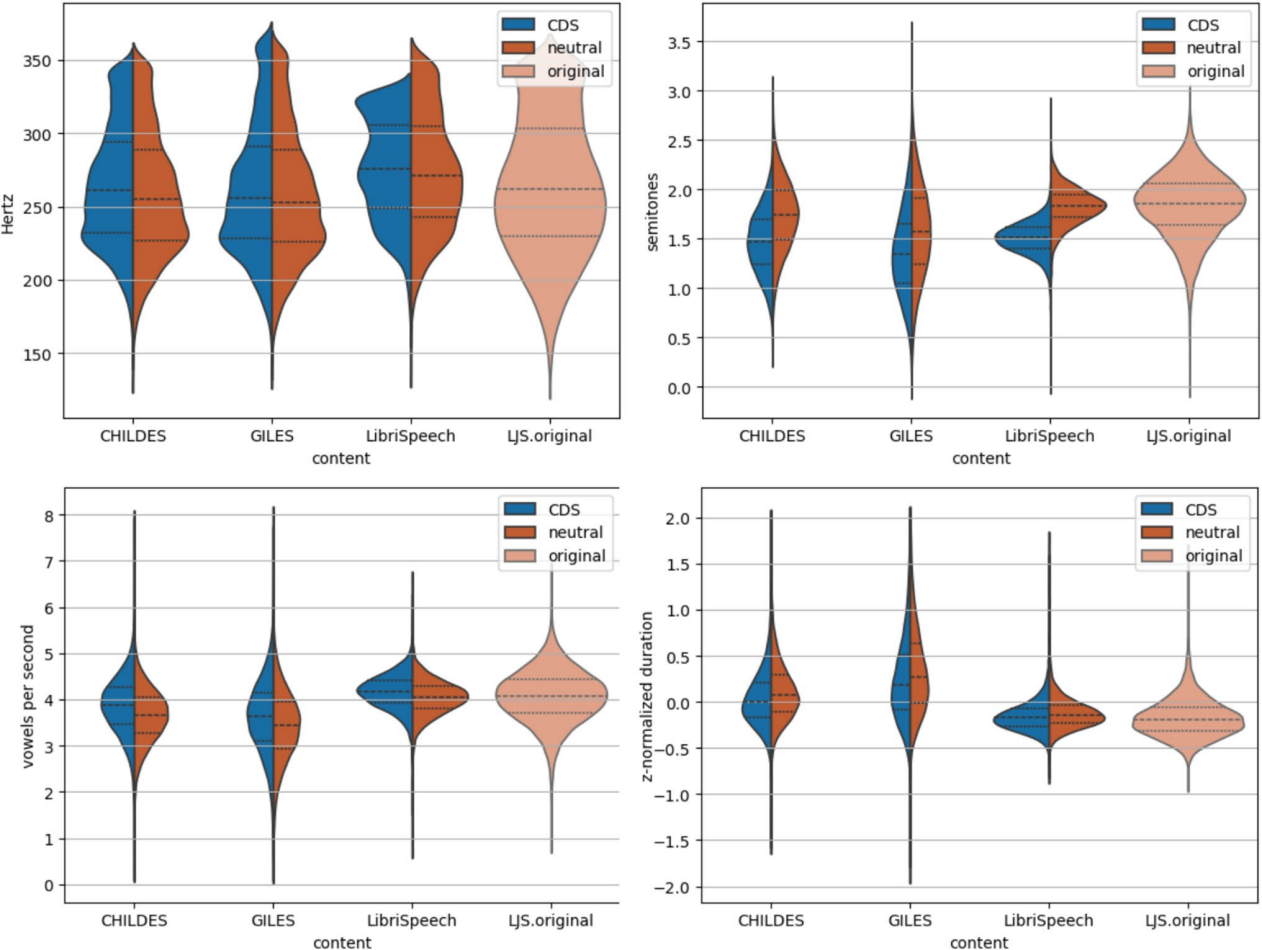
Results of the prosodic analysis as a function of different linguistic speech content (*x*-axes) and speaking styles (synthetic CDS = blue, synthetic neutral = red, original LJSpeech = light red). The violin plots show the distribution of acoustic measurements across all analyzed utterances. Top left: *F*_0_ of the first word accent in the utterance (in hertz). Top right: *F*_0_ standard deviation within the utterance (in semitones relative to the *F*_0_ mean in the utterance). Bottom left: number of vowels per second. Bottom right: *z*-score-normalized duration of vowels

Figure 5 shows the results from the prosodic analysis, where the goal was to identify the effects of text content (books versus CDS transcripts) and speaking style (neutral versus CDS) on the resulting synthetic speech, as the aim was to have control over the generated speaking styles.^6^ The speech produced with a neutral speaking style has a slightly lower pitch (Fig. 5, top left) and greater variability (Fig. 5, top right). The greater variability might be explained by the much larger training data for this style (30 h of neutral speech vs. 1.1 h of CDS speech) and thus greater variability in the melodic properties of the training speech data. However, manual listening also revealed that the original speaker of the LJSpeech corpus, the speaker used to train the neutral voice, sometimes used intonation and prosodic marking in a highly expressive manner while reading the audiobooks, which might have also contributed to the high variability of pitch in the synthetic data. As for other measures, the speech produced with CDS style has a slightly higher tempo (Fig. 5, bottom left) and shorter vowels (Fig. 5, bottom right). This is likely because the CDS contained conversational speech only, while the neutral speech data were mainly carefully read speech.

For linguistic content, the conversational speech (CDS content from CHILDES and GILES) is produced at a slower tempo (see Fig. 5, bottom left) and with longer vowels (Fig. 5, bottom right) than the book content (LJSpeech original and Librispeech synthesized). The *F*_0_ value does not seem affected by the linguistic content, although the variability of utterance-level *F*_0_ standard deviation is larger for the conversational speech contents. Note that the content is not explicitly conditioned during the TTS generation, but it influences the output due to differences in text content.

We also performed statistical comparisons for the prosodic measure distributions. After the Shapiro–Wilk test revealed non-normality of the distributions, the Wilcoxon signed-rank test was used to conduct pairwise tests for similarity of the distributions. The test showed that there was a significant difference between all pairs of compared conditions (speech content, speaking style) (*n* > 5,978, w > 735,566, *p* < 0.05), except for *F*_0_ of the first word accent in an utterance when comparing CHILDES and GILES content generated with a neutral style (*n* = 19,011, w = 88,213,990, *p* = 0.306).

Overall, the results show that both the selected speaking style and linguistic content of speech influence the prosodic properties of the generated speech. This demonstrates that synthetic speech generation can incorporate different speaking styles and behaves differently depending on the type of speech content, allowing controlled variation in speech properties when GILES is used for data generation.

### Interim summary

The results of the data generation showcase the ability of the LM to capture linguistic properties of CDS at a transcript level, and the ability of the TTS system to capture different speaking styles through explicit style conditioning and by distinguishing between how book contents and conversational utterances are spoken. Moreover, the results show that the system generates novel CDS utterances that are not attested in the training data, thereby also resulting in novel phonetic forms (coarticulatory contexts and phonotactic patterns) that would not occur in re-synthesis of CHILDES alone. While the current implementation of the pipeline is far from reaching perfect CDS in terms of content and still lacks a proper CDS speaking style across many different voices, the results act as a proof of concept for GILES as a means of creating controllable training data for computational simulations. In addition, the metrics employed clearly identify areas for improvement in future work.

## Experiment 2: Language learning simulations with the synthetic data

The aim of the second experiment was to test whether computational language learning simulations are impacted by controlled changes in the speech input, as produced with the GILES pipeline. If this is the case, this would demonstrate the importance of paying careful attention to the data used in modeling experiments and the potential of GILES as a means to study such variation. To this end, we trained an SSL-based learner model on various alternative speech datasets by manipulating the type of linguistic content (audiobooks vs. CDS transcripts) and speaking style (neutral vs. CDS expression) while keeping the total amount of language exposure the same. As an outcome measure, we tested the model for its ability to discriminate native phonemic contrasts and to categorize early vocabulary word forms during the learning.

### Learner model

As a computational model of an infant learner, we employed a learner model based on contrastive predictive coding (CPC; van den Oord et al., 2018) that operates on acoustic speech input in an unsupervised manner. The same learner model was successfully used in several earlier studies, where it demonstrated the ability to learn phonemic contrasts and rudimentary word-form discrimination for native language vocabulary (e.g., de Seyssel et al., 2023; Lavechin et al., 2023b), but also replicated increasing preference toward IDS speaking style with more language experience (as observed empirically in ManyBabies; Cruz-Blandón et al., 2023).

In short, CPC can be viewed as an artificial neural network implementation of a statistical learner (see de Seyssel et al., 2023, for further discussion of the analogy), where the aim of the learner is to correctly predict future evolution of a speech signal, given access to the speech input up to the present moment. Instead of operating on some predefined linguistic representations, such as learning transition probabilities of syllables (cf. Saffran et al., 1996; Frank et al., 2010), the model operates on raw acoustic waveforms, making it applicable to real speech without a priori linguistic assumptions or constraints. Moreover, instead of predicting the speech waveform itself, CPC learns to predict its own (latent) representations encoded from the speech signal while simultaneously learning these representations. This ability to learn its own prediction targets allows the model to abstract away from minute details of the acoustic input, focusing on aspects of the input that are predictable over time, such as phonetic structure and prosody of the signal.

Although the CPC learner has been demonstrated to learn phonemic contrasts and signs of proto-lexical organization (see above), in this work we do not wish to make the case that CPC would somehow be a good model of an infant learner. We simply use it as a well-established and publicly available model capable of using real speech as input, with the overall aim of testing whether controlled differences in language exposure map to different learning outcomes.

In our experiments, we used the CPC implementation of Lavechin et al. (2023b), which is based on the implementation of Rivière et al. (2020) and was also used as a baseline system in the Zero Resource Speech challenge 2021 (Dunbar et al., 2021). The CPC model consists of an encoder and temporal modules, where the encoder of the present implementation is a five-layer convolutional neural network (CNN) with gradual downsampling, responsible for mapping the waveform (sampled at 16 kHz) into 256-dimensional latent embeddings occurring every 10 ms. The temporal module is a recurrent neural network (RNN) layer consisting of 256 long short-term memory (LSTM) cells. At each time step, the RNN sees the past encoder latent vectors **z**_*t*_ up to time *t* and accumulates the information into a context-sensitive embedding **c**_*t*_. This embedding is then fed to a transformer layer with self-attention that maps the context-sensitive RNN output into a set of predicted future latent vectors **z**_*t*+1_*, ***z**_*t*+2_*, …, ***z**_*t*+*K*_ up to a maximum distance of *K* = 10 frames (100 ms). The network is trained using a contrastive loss function aimed at maximizing the similarity of predicted and true future latent states while minimizing the similarity of predicted latent states and randomly sampled true latent states from other time steps (see van den Oord et al., 2018, for details). When evaluating the model’s learning outcomes, we extracted the context-sensitive RNN outputs **c**_*t*_ for each frame and analyzed them in terms of their ability to represent linguistic content (see the next subsection).

### Experimental setup

To study bootstrapping of phonetic and lexical learning, we created six alternative training sets of approximately 100 h of speech using the datasets and GILES components described earlier, as previous research has indicated substantial phonetic learning and above-chance lexical learning already at that amount of speech exposure (de Seyssel et al., 2023). These sets follow the conditions of Experiment 1, where contents of the speech could be book contents (from LibriSpeech), original CDS transcripts from CHILDES, or CDS transcripts generated with GILES. All three content types were then synthesized into speech in the neutral and CDS speaking styles.

For CHILDES, we accumulated data from the youngest recipient ages until reaching the total word count of the reference 100-h GILES dataset, i.e., from 6 to 24 months. GILES data were generated by producing approximately 50 h of speech with 6-month-old recipient conditioning, 25 h with 9 months and 25 h with 12 months. Since the speaking rates of different styles and contents vary slightly, not all datasets were exactly 100 h in duration. CPC training was then conducted independently on each of the datasets using a fixed random seed to ensure identical initialization of the models. Training was run for 50 epochs using the Adam optimizer (lr = 0.002; β_1_ = 0.9, β_2_ = 0.999, ε = 10^−8^) while monitoring the linguistic competence of the learner during the training.

The models were tested on two basic early language skills: ability to discriminate phonemic contrasts and ability to discriminate acoustic word forms. For phonemic discrimination, the widely utilized ABX-test was employed (e.g., Schatz et al., 2013, 2021; Dunbar et al., 2022) using the latest implementation of the test from the ZeroSpeech toolbox (http://www.zerospeech.com). The ABX-test probes the model’s ability to discriminate phonemic contrasts in minimal pairs (e.g., “*bat*” vs. “*bit*”), where a 50% ABX error rate indicates chance-level discrimination and 0% indicates perfect discrimination (see Schatz et al., 2013, for details). Acoustic word-form discrimination was tested using the CDI-Lextest from Khorrami et al. (2023; https://github.com/SPEECHCOG/CDI_lextest/). In CDI-Lextest, the model is fed 89 different isolated words typical of early infant receptive vocabulary (list extracted from CDI WordBank; Frank et al., 2016), each synthesized in 20 different voices. The corresponding latent word representations of the model are then analyzed with respect to how well they cluster into word types in terms of cosine distance. A test score of 100% indicates perfect lexical categorization, while chance level is slightly above 1%. The test can be performed using either frame-level or word-level representations (multiple embeddings vs. only one embedding per isolated word), and we used the latter as it better focuses on lexical instead of phonemic competence of the model. For this purpose, we calculated the average of the CPC’s context embeddings **c**_*t*_ across all the time steps *t* in the input as the word-level representation.

We report the performance of these two tasks as a function of training time (epoch), focusing separately on the effects of speaking style and speech content. When focusing on style, average performance across the three content types will be reported, and when focusing on content, average performance across the two styles will be reported. Baseline performance before any training is also reported as a reference for both tasks.

### Results

**Fig. 6:**
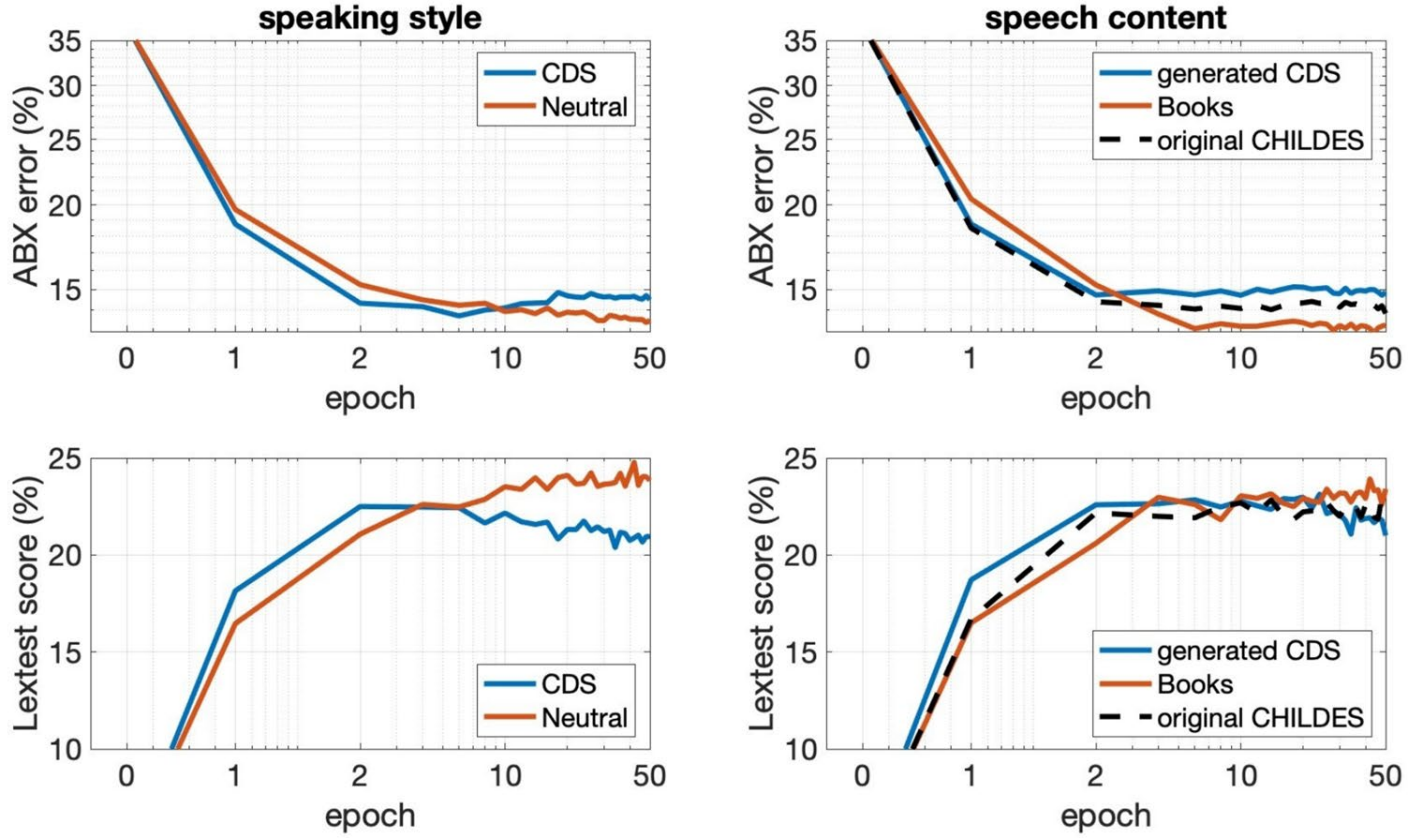
Results from the computational simulations as a function of training epoch. Top: phonemic minimal pair discrimination error from the ABX-test (%; lower is better). Bottom: lexical discrimination score from CDI-LexTest (%; higher is better). Left panels: more expressive (blue) versus neutral (red) prosody. Right panels: CDS (blue) vs. book content (red). The results shown are averages across the other non-compared data factors

Results of the learning simulations are shown in Fig. 6. Before any training, CPC with random initial weights achieves an ABX error rate of 36.82% and LexTest discrimination score of 4.31%, which are slightly better than the corresponding chance levels.

The first key finding is that all generated datasets result in systematic gradual improvements in phonemic and lexical competence over learning epochs, thereby demonstrating that the synthetic data from GILES can be used to investigate and compare models’ learning capabilities in different linguistic skills. In terms of learning conditions, it appears that learning is always initially faster with CDS prosody than with neutral prosody. Similarly, synthetic CDS speech content results in faster learning compared to the same amount of linguistic content from books. The advantage in learning speed is notable for both metrics. For the CDS prosody, close-to-best phonemic and lexical scores are already achieved on the second epoch (~200 h of speech if iterative training is counted as gradually increasing exposure), whereas neutral prosody reaches comparable performance several epochs later. Likewise, on CDS speech content, close-to-best phonemic and lexical scores are already achieved on the second epoch, whereas audiobook content requires 1–2 additional epochs to reach comparable performance levels. However, the final performance scores are somewhat higher for neutral prosody and book content. This is paralleled by signs of slight model overfitting emerging during later epochs for the CDS prosody and CDS content. This suggests that the model, with its hyperparameters (modeling power) initially designed for larger-scale multi-speaker data, might start to become too specific to the variability inherited from limited-size training data. Importantly, both phonemic and lexical learning are comparable between GILES-generated CDS content and the original CHILDES transcripts, indicating that the synthetic linguistic content does not degrade learning performance compared to real empirical data.

Overall, the results show how a more expressive speaking style with simplified syntax and lexical content of CDS results in faster learning relative to linguistically more complicated book contents or less expressive speaking style. This corroborates earlier findings from LM training in Huebner et al. (2021), where the authors showed how CDS transcripts resulted in faster learning of syntactic knowledge than a comparable amount of Wikipedia texts, on average.

Note that the TTS voice used in the current study was only present in the audiobook part of the training data (LJSpeech), which consisted solely of neutral-style read speech, whereas the “CDS” style was only spoken by the speakers of the EXPRESSO dataset. The speaking style and the spoken content not only show up in acoustic measures (Section"Experiment 1: Evaluation of the synthetic data"), but also result in observable differences in learning outcomes in the simulation, including faster learning. In other words, the advantage of CDS content and CDS style outweighs the potential benefit of higher-quality TTS from matching style and content in the initial stages of speech learning from the synthesized input.

Also note that the absolute scores are still substantially lower than those observed for previously reported studies with real speech, such as the ABX scores in the ZeroSpeech challenge (see the leaderboard at http://www.zerospeech.com). However, the present data consist of only one voice, whereas previous studies have used corpora with several (up to hundreds) of speakers, which is likely to provide a major advantage in the present benchmarks, where the model is tested on speech samples from numerous different speakers. For the present purposes, the aim was not to demonstrate state-of-the-art performance, but to show how different input factors contribute to learning outcomes when tested in a controlled manner.

## Discussion

Computational simulations of early infant language development must be based on learning from speech input, as the acquisition process and nature of the speech representations utilized by infants are still largely unknown. Instead, they constitute some of the key questions to be addressed by modeling research (see also Dupoux, 2018; Lavechin et al., 2022). Despite the recent increases in audio data availability from naturalistic infant surroundings (e.g., Cychosz et al., 2020; Demuth et al., 2006; Gilkerson et al., 2017), infant head-mounted cameras (e.g., Bergelson et al., 2019; Long et al., 2024), and crowdsourced read speech corpora (e.g., Panayotov et al., 2015; Harwath et al., 2016), the research community is still lacking representative, controlled, and high-audio-quality speech corpora to simulate infant language experiences at a realistic scale through the first years of life. Moreover, no existing corpora enable systematic and *controlled* modeling studies on how different factors in language input impact learning outcomes, while also maintaining the representativeness and realistic scale of the input. This is also due to the fact that real-world language (and sensorimotor) experiences of infants always vary in countless different ways, preventing the collection of several longitudinal, dense, and real-world audio corpora that truly differ by only one factor of interest at a time.

In this paper, we have presented one strategy for conducting controlled language acquisition simulations. The idea behind the GILES pipeline is to synthetically imitate the speech input that infants of a desired age range would hear during their development, and to do it as accurately as possible by making use of what is known about infant language experiences. At the same time, statistical connections between speech input and extralinguistic factors affecting the input, as present in empirical data, can be used to control the generation process, enabling simulation of the potential effects of these factors on learning from speech data. Naturally, unrealistic or hypothetical input scenarios can also be simulated, given that they are implemented as a part of the stochastic generators or as post-processing steps to the data. By comparing generated data against real data on infant input, limitations of the approach can be made transparent and corrected in later versions of the pipeline.

In our experiments, we tested a proof-of-concept implementation of GILES in its ability to create realistic CDS transcripts and to synthesize those to speech in different speaking styles, showing success in both despite the relatively limited training data available for system training in these tasks. In addition, we replicated earlier computational learning experiments in phonetic and lexical learning using a CPC-based learner model, further showing how GILES can be used to control the properties of the generated data, and how this maps into different learning outcomes in the learning process. While the current GILES implementation and the associated experiments are still limited in many ways (see the next subsection), the present work provides evidence for the feasibility of the concept.

Naturally, the use of synthetic data is no magic bullet. Given the current machine learning technologies, it is well known that even large statistical models fail to reproduce the full distribution of the original training data (e.g., Dohmatob et al., 2024)—a problem that is likely to be exacerbated in power-law-like distributions with long shallow tails typical in language. Similarly, the neural models generally struggle to extrapolate beyond their training distribution (e.g., Marcus & Davis, 2019). However, at the same time, the existing statistical LMs and TTS systems show that they can create linguistic and phonetic content that goes beyond their training data, such as learning the generative compositionality of language (e.g., Vaswani et al., 2017; see also Fig. 4), generalizing coarticulatory phenomena to such new content (at least to some accuracy), or transferring speaking styles and prosodic patterns to linguistic content never observed at training time (see Triantafyllopoulos et al., 2023, for a review), even when trained on relatively limited data, as was the case in the current proof-of-concept study. When additional relevant data resources and improved LM and TTS methods are incorporated, the amount of variability captured and reproduced by these models will increase.

Overall, we see the use of synthetic data as complementary rather than a substitute for other approaches that make use of different types of speech (and multimodal) resources for modeling infant learning. Synthetic data provide the advantage of control over the quality and quantity of data, and can be called representative in terms of measurable properties that are statistically indistinguishable from empirical data. Yet, it is never real infant input, and as long as we lack complete longitudinal characterizations of speech input to infants across their early life, it is impossible to say whether and how the synthetic data deviate from the real-world input.

### Limitations and future work

The main conceptual limitation of GILES is that the generator will, by default, inherit all the limitations and biases of its training data. If the original data are not naturalistic or at least partially representative of the target audience, the generated data also cannot be. If the training data do not contain sufficient variability in those properties of CDS that are relevant to control in the intended learning simulations, the pipeline cannot create speech data that vary along the dimensions of interest. Ultimately, this means that the representativeness of GILES data is always limited by the availability of real-world CDS data to train it. Moreover, although modern ML systems can model rich variability in input, there is always some loss of information between the source data and its statistical model (cf. Dohmatob et al., 2024). However, compared to the alternative of just directly using the existing CDS datasets as they are, the primary advantages of the approach still remain: *generativity* in terms of creating novel speech content and acoustic forms *beyond* the training data, *controllability* in terms of different aspects of quality and quantity that are of interest to researchers*, data anonymity* through stochastic processing, and *transparency* in terms of what the created data look like through rigorous measurement. This means that the GILES approach enables certain types of modeling research that are difficult to conduct otherwise, but it does not replace real speech data for cases where suitable naturalistic data in terms of quality and quantity are available to address the questions of interest.

The current practical implementation of GILES also comes with several related limitations. First, the vocabulary generated by the LM is somewhat smaller than that of the original data (as indicated by TTR) and as predicted by theoretical and empirical considerations (see Dohmatob et al., 2024). Moreover, CHILDES, despite being the best CDS transcript resource available, is still very limited in size compared to typical datasets used to train contemporary LMs. In post hoc tests, we explored different sampling temperatures to add more variability to the vocabulary. While higher temperatures increase TTR to a CHILDES-compatible range, they also result in more syntactic errors, which increase the perplexity beyond the empirical range.

The present work also did not evaluate whether the linguistic properties of the data scale up realistically for larger datasets totaling thousands of hours. For instance, the vocabulary of the LM is largely determined by the vocabulary present in CHILDES (although new words are invented at times; see the new one-word rates in Fig. 3), whereas real caregivers will have a broader vocabulary of English at their disposal. Since the intended use case is to use GILES to produce ecologically plausible CDS on scales beyond the size of CHILDES, future work should explore additional techniques for vocabulary enrichment to ensure the accuracy of TTR and the realism of data size scaling. These could include, for instance, original and synthetic data mixing (cf. Dohmatob et al., 2024), injecting additional vocabulary at training time or as a post-processing step, or via inclusion of additional training corpora such as those in the BabyLM Challenge datasets (Warstadt et al., 2023). In general, scaling law issues in the context of synthetic data (Dohmatob et al., 2024) should be carefully analyzed and addressed in our particular use case.

As for the TTS, the main limitation is that there is no existing system for producing naturalistic CDS despite the otherwise major recent developments in the field of speech synthesis, likely due to a lack of suitable datasets for model training. We also failed to train TTS using CDS audio from the Providence corpus (Demuth et al., 2006), as the sound quality in the data was not sufficient for reaching an acceptable model for FastPitch (Łańcucki, 2021). A similar problem appears when trying to use reference encoder techniques, such as XTTS (Casanova et al., 2024), to transfer prosody of CDS samples to generated speech, as these TTS systems also tend to copy the (poor) sound quality of the reference recordings to the generated output. For the current GILES, this means that the generated speech is not proper CDS in prosodic terms, and that the speaking style of the TTS does not follow recipient age-dependent patterns even though the content does. In future work, multiple high-quality voices, ideally capable of both ADS and CDS, will be needed for more accurate reproductions of infant language experiences. To achieve this, new techniques for style transfer or prosodic conditioning from noisy CDS data to TTS voices trained on clean audio would have to be developed. Nevertheless, TTS technology is rapidly evolving, and powerful LLM-based TTS systems capable of controlling speaking style and speaker identity have started to appear (Casanova et al., 2024). Thus, we believe that in a few years, better TTS systems capable of copying CDS speaking style from examples will emerge and will then be incorporated into the current framework.

Experiment 2 also has several limitations, some of them originating from the high computational demand of the learner model employed. First, the amount of speech data was limited to 100 h per condition, as CPC model training in each of the conditions still took several days despite a relatively powerful GPU. In contrast, data generation for these simulations was much faster even when performed on a laptop computer. Moreover, the simulation did not adequately address incremental (one-pass) learning despite our arguments in favor of such an approach (see Introduction), as the current neural network-based models are not designed to learn from individual exposure to data points. In general, the simulation simply showcased the impact of two factors: speech content and speaking style. In practice, there are countless questions that can and should be addressed with GILES, ranging from variation in linguistic input to differences in speaker distributions, amounts of speech exposure, importance of age-dependent CDS properties, and many other factors of interest and relevance. Some of these will require extension of GILES with new capabilities, such as inclusion of new TTS voices to model different speakers or new controllable factors in the text or speech generation (e.g., modeling individual differences in the lexical, syntactic, or phonetic properties of caregivers’ speech). Some others (e.g., the amount of speech exposure, age-appropriateness of linguistic form) can already be addressed in its current form. Note that many of these extensions are expected to be quite straightforward to incorporate in the GILES pipeline in the form of conditioning the LM or TTS systems. They will simply require some manual effort and suitable training data in which the relevant variability of interest is expressed.

In general, the purpose of the present experiments was to demonstrate the GILES concept by controlled manipulation of some key variables in the language input (here: linguistic content, speaking style, speaker identity). In practice, infants are exposed to a much richer distribution of various types of speech, for example, in terms of style, content, intent, speaker, and hence the present results should not be treated as attempts to simulate learning in a realistic manner. Instead, given the present conceptual framework, future studies can investigate the effects of different input factors on language learning at different levels in a controlled manner. By first obtaining empirical estimates for the variability of different factors, such as the proportion of IDS and ADS that infants in different families are exposed to, corresponding training datasets can be created and experimented with.

Obviously, all the research discussed in the current context focuses on the learning of language from (passive) exposure to audio input, and therefore does not cover the full breadth of phenomena involved in learning. For instance, infant–caregiver interaction adapted to infants’ needs and developmental level, active exploration, multimodal perception, and concurrent development of speech production are not considered here. This is a deliberate limitation, focusing the present contributions on the relatively established practice of studying language learning from speech only. However, the approach could be extended to more interactive settings or to support multimodal simulations in the future.

Finally, the first experiment reported a broad range of linguistic properties that appear to change with the recipient infant age in empirical CDS data. However, it should be noted that the CHILDES database used is a heterogeneous collection of CDS corpora collected from various settings (lab vs. home, free play vs. scripted tasks). This may impose different biases to the type of CDS in each of the age bins. However, the studied age bins contained data on average from 10.6 different corpora (min 2, max 17), which mitigates the effects of corpus-specific factors. This is also indicated by the relatively smooth age-dependent trends of the linguistic measures. Nevertheless, future work should replicate the analyses with statistical models that control for the potential known factors of the individual corpora.

## Conclusions

Ecologically plausible, and thereby useful, computational models of early language acquisition require good models of the learner, learning outcomes, and the learning environment (see also de Seyssel et al., 2023). This work proposed a framework for the environment modeling, namely speech input modeling, using a statistical stochastic generation with a pipeline called GILES. We showcased how GILES is able to model age dependencies of linguistic properties of CDS and synthesize the generated caregiver utterances into speech audio in different speaking styles. Our experiments with computational learning simulations also exemplify how GILES can be used to study the impact of various factors affecting the speech heard by the learner. Together these experiments provide initial proof of feasibility for the approach.

Yet, much work is to be done in order to create a more representative and comprehensive model of the infant language exposure, including individual variation in experiences, in order to use such data in computational learning experiments—be it synthetic or real recordings. In the long term, we believe that the research community would benefit from standardized yet flexible model training and evaluation protocols that would enable replicable modeling experiments with shareable data, thereby supporting the comparison of alternative (computational) explanations of the learning process and the accumulation of scientific understanding through shared practices and assumptions. We hope that GILES provides one starting point towards such common practices, and that further development of an open-source GILES will continue in the years to come in a collaborative manner with the broader computational modeling research community.

Naturally, all potential studies making use of GILES are ultimately dependent on the learning models employed, where we currently lack models capable of fitting infant data across a broader range of early language phenomena and across the developmental timeline (Dupoux, 2018; Cruz Blandón et al., 2023). However, given the possibility for controlled modeling studies using GILES together with the existing training paradigms with real prerecorded data, and combined with a rich array of evaluation metrics to compare models to human learning (Cruz Blandón et al., 2023; Lavechin et al., 2023b; Tan et al., 2024), the community can work towards models that are increasingly capable of explaining early language phenomena more broadly.

## Data Availability

The training data used for the text generator are publicly available from CHILDES database https://childes.talkbank.org/, and the LJSpeech and EXPRESSO speech data used for speech synthesis system training are openly available from https://keithito.com/LJ-Speech-Dataset/ and https://github.com/facebookresearch/textlesslib/tree/main/examples/expresso/dataset, respectively. None of the data and materials developed for the reported research were preregistered.

## Appendix 1: Example CDS transcripts generated with the GILES

Below is a list of example generations from the LM trained on CHILDES data, as conditioned with different infant ages. Each bullet point corresponds to a separate random seed prompt to start the generation. Note that all punctuation was replaced with full stops for the LM training and all transcripts were lower-cased, and thereby the generated texts inherit these properties.

### 6-mo

- yay. yay. yay. yay you're my cutie cutie cutie. silly nose. ahhah. there's a bird house. mama. oh did you say good jo. you're my. think a bird's gonna get all messed up. ahhah. yay.
- on. yuck. yes we are. yeah. bang bang. we'll go to the ball. okay right. you missed again. uguh. oh it gets silly today. bang bang. let go. gonna play with your car. very good.
- mommy helps you. oh now doesn't wanna have a cookie. uhoh. just one of those big birds. mommy help you. mm. lots of birdies go there. come on. man. one again.
- these are tools but then they'll probably go together. because you wanna trade lots of shapes. let's see. well just look at this. hm. oh for mommy. no no. come on. hey theo. there you go. yeah.
- see it. yeah. go get it. quit fussing for a boy. yeah. who are you gonna get it. are you gonna get it. good jo. very good. alright. yep. is that your little stool. oh. you're gonna get them. yeah a.

### 12-mo

- a baby clown. yeah. we're both trying to get it going. a baby baby. yeah. that's right. come on. oh. little ducks. ball. there we go. there we go. it's kinda funny. let's see.
- boy lemme see. baby. big bird. oh the kitty. what um. hey. alex fix a soft string for me. and close. owl. you got it again. sure. let's try something else.
- you move your leg. so you don't slip huh. timmy's shirt. come on. you wanna finish up with your pee pee on your chair. what's gonna happen. can you wipe your bottom. clean up.
- come on. it's a sheep. yay. yay joseph. look at this beautiful sheep. huh yay yay. whoa. uh whoa. that's kinda silly looking at it. what's that. what's that.
- you're spitting up the cereal. get out. where did your belly button. whoopsie. are you being real real to me. no no. xavier don't play. don't play with that. now you take a shower. no.

### 18-mo

- peek a boo. bunny's gonna like a chick doggy. choo. jack inside the box. what's this. uhhuh. is that a duck. quack quack quack quack. who's that. does that come off the page. oh look at the duckies.
- uh what. what do you have on your eyes. where's big lam. where's big tail. where's big lam. is that big lam. where's big lam's eye. where's big tail.
- a magnifying glass. show me. you can sit in your chair. oops. there you go. uhoh. got it. tell me what this is. uhhum. oh where it went. uhhum. what about this. you ain't got it from about ten cents.
- how about the pig. see the pig. here let's wipe what else. too straight. whats this. see. see the doggie. yeah. can you say paul. you think so. thank you. johnny. pen.
- mommy's gonna dry your dishes. a bowl yeah. yeah me get your cup. baby's gonna wash your bottom. baby's sleeping. oh. show mommy what you ate. wash your hands.

### 48-mo

- okay. you can see yourself later. look at this. one piece again. no. what happened when you do batman. oh you're gonna hold it too. put it right up. oh. now.
- okay. and then we can use the whole deck of our tower. you're just gonna do it right now. you hafta order something. yeah. we can fly in texas. alright you should stand on the couch and do it. have you ever seen a ghost outside tonight.
- yes. we'll ask karen if she'd wear it. what cha doing to your stomach. yes. would someone ever talk about a bottle of milk. yep. that's super nice and hungry. do we need to cover up books.
- it's in the way where we gonna wash them. wanna do it again. huh. you did though. oh yeah i did. did you see some pictures. okay. you set her up for a long time. we could save some for mom to finish ya.
- no i don't remember where a couple of em is. okay. what. oh boy. what's the matter. what. okay. nah i thought about the boys're getting really fresh when you went to the park. oh. what's this.

## Footnotes

1 E.g., does the input have to be IDS? How many hours is the baby awake per day? What is an acceptable signal-to-noise ratio? In what culture/language environment?

2 Here we focus purely on representativeness of the speech exposure, but acknowledge that many other factors, such as interaction, multimodal input, and caregiver feedback, are likely to affect the learning process and hence the *representativeness of the simulations broadly.*

3 The development of a high-quality system for CDS TTS is a nontrivial problem due to lack of representative large-scale speech data that is of sufficient audio quality for TTS model training.

4 https://huggingface.co/openai-community/gpt2

5 https://github.com/librosa/librosa

6 Note that direct comparison against real CDS recordings was not possible, as the TTS system aims to capture speaker-independent prosodic properties of CDS from the training data instead of imitating the speech of any specific speaker.

## Code availability

All program code used in the data generation and analysis experiments is publicly available at https://github.com/SPEECHCOG/GILES_pilot.

The FastPitch TTS system used in the experiments is available at https://github.com/NVIDIA/DeepLearningExamples/tree/master/PyTorch/SpeechSynthesis/FastPitch. The CPC model and the ABX-test used for computational modeling experiments are available at https://github.com/MarvinLvn/CPC2, and the CDI-Lextest is available at https://github.com/SPEECHCOG/CDI_lextest.
